# Detecting Pattern Changes in Individual Travel Behavior from Vehicle GPS/GNSS Data

**DOI:** 10.3390/s20082295

**Published:** 2020-04-17

**Authors:** Jingfeng Lou, Aiguo Cheng

**Affiliations:** State Key Laboratory of Advanced Design and Manufacturing for Vehicle Body, Hunan University, Changsha 410082, China; loujingfengqdu@163.com

**Keywords:** pattern change detection, individual travel behavior, vehicle GPS/GNSS data

## Abstract

Although stable in the short term, individual travel behavior generally tends to change over the long term. The ability to detect such changes is important for product and service providers in continuously changing environments. The aim of this paper is to develop a methodology that detects changes in the patterns of individual travel behavior from vehicle global positioning system (GPS)/global navigation satellite system (GNSS) data. For this purpose, we first define individual travel behavior patterns in two dimensions: a spatial pattern and a frequency pattern. Then, we develop a method that can detect such patterns from GPS/GNSS data using a clustering algorithm. Finally, we define three basic pattern-change scenarios for individual travel behavior and introduce a pattern-matching metric for detecting these changes. The proposed methodology is tested using GPS datasets from three randomly selected anonymous users, collected by a Chinese automotive manufacturer. The results show that our methodology can successfully identify significant changes in individual travel behavior patterns.

## 1. Introduction

Understanding individual travel behavior is important in commercial advertising, location-based service (LBS) design, travel demand management, and urban planning [1]. However, existing work on travel behavior modeling often makes the implicit assumption that a person’s travel patterns are stable [2]. Although stable in the short term, individual travel patterns are subject to change over the long term. Individual travel behavior may change when people change jobs, move, purchase a new car, shift work schedules, or change their travel and activity habits as a result of other events (e.g., when a child starts school). For example, when people move from the suburbs to the city center, they may shorten their travel distance, increase their overall travel frequency and the number of locations they visit, and shift their commuting hours.

Previous research on travel pattern changes has focused on the effect of economic, environmental, social, and attitudinal factors [3,4,5,6], as well as ways of using these factors to induce changes in travel patterns [7,8]. Zhan et al. [2] proposed a method to detect whether a pattern change has occurred and, if so, to identify the time points of such changes, referred to as changepoints. However, no attention has been paid to how individual travel behavior changes. Understanding and adapting to changes in individual travel behavior is important for product and service providers in a continuously changing environment [9]. For example, by identifying when users change the places they frequently visit, automotive manufacturers can discover new vehicle usage scenarios and can thus infer new user requirements and make product adjustments.

Current research in the context of change mining is limited to customer purchase behavior research available in the literature [10,11,12,13,14]. Relatively little attention has been paid to mining changes in individual travel behavior from vehicle GPS/GNSS data. Furthermore, the pattern mining methods typically used to detect changes in purchase behavior are decision trees and association rule mining technology [15], which are unsuitable for directly mining travel behavior.

In this paper, we propose a clustering-based method to detect how individual travel behavior changes. For this purpose, we first define two dimensions of individual travel behavior patterns: spatial and frequency patterns. Then, we develop a method that can detect such patterns from an individual’s GPS/GNSS data by using a clustering algorithm. Finally, we define three basic pattern-change scenarios for individual travel behavior and introduce a pattern-matching metric to detect these changes. The remainder of this paper is structured as follows: in Section 2, we provide an overview of related work. Section 3 introduces the proposed methodology in detail. A case study is reported in Section 4 to illustrate the application of our technique. In Section 5, we conclude the paper and discuss future work.

## 2. Literature Review

### 2.1. The Study of Travel Behavior

Early travel behavior models mainly adopted the classic trip-based method, commonly referred to as the four-step model, which originated in the United States in the 1950s to evaluate the impact of building infrastructure on travel behavior. However, because the four-stage approach oversimplifies the travel-chain process and is incapable of providing a microscopic analysis of human behavior, Mitchell and Rapkin [16] proposed an activity-based approach that relies on the decision-making process involved in people’s travel behavior. Many scholars have made significant contributions to the development of this theory. Hägerstrand [17] developed a time-geographic approach that described the systems of constraints on activity participation in time-space. Chapin [18] proposed a dynamic framework that identified patterns of behavior through space-time. Fried, Havens, and Thall [19] addressed social structure and the question of why people participate in activities. Travel links the places where people go to meet their obligations and lead their lives.

Traditional travel diaries or travel survey data were mostly derived from household interviews or regional censuses [20]. Over the past decade, with the development of data collection from transit smart cards, GPS/GNSS [21,22,23], and mobile phones, direct travel trajectory data have been complementing or replacing conventional travel data at a rapidly increasing rate. These data help us to understand human travel behavior better by zooming into individuals’ behaviors more closely than ever. Ciyun Lin et al. improved the traditional Motor Vehicle Emission Simulator model by adding real-time GPS datasets, and the results showed that the model could effectively improve the estimation accuracy of traffic emissions and provide a strong scientific basis for environmental decision-making, planning, and management [24]. Consequently, various data mining techniques have been proposed to gain insight into travel behavior from trajectory data. Trajectory data mining can be classified into several categories, including pattern mining, clustering, classification, and prediction [25]. Trajectory pattern mining aims to discover and describe travel patterns hidden in trajectory data. It provides information about where and when patterns occur and identifies the entities involved. A review of many types of travel patterns can be found in the literature [26]. One branch of research [27,28,29] considers the problem of trajectory clustering. It is interesting to cluster trajectories into groups with similar patterns. Groups of people can then be identified from trajectory-related information (e.g., temporal duration, spatial dispersion, movement velocity) as well as the semantic meaning of locations. Trajectory classification aims to identify the class label of trajectories from a predefined label set [30]; for example, it can identify the mode of travel on the basis of trajectory features. Prediction mainly seeks to infer a person’s future location on the basis of existing trajectories [31]; this approach has been especially motivated by the fast-growing development of LBSs, one of its major application areas.

Data mining is the process of exploring large quantities of data in order to discover meaningful patterns. However, much of the existing data mining research has focused on a static picture describing the composition of the dataset. Little attention has been paid to mining pattern changes.

### 2.2. Mining Changes

Scholars have developed many methods to recognize changes between databases. The first study of change mining stemmed from Liu et al., who developed an approach to change mining that uses decision trees to predict changes in customer behavior. Song et al. [9] proposed a methodology that automatically detects changes in customer behavior via association rules from databases at different time periods. Chen et al. [13] integrated demographic variables, customer behavior variables, and transaction databases to establish a method of mining changes in customer behavior. Cho et al. [14] proposed a new methodology for enhancing the quality of collaborative filtering recommendations that uses the evolution of customer purchase sequences over time. Huang, Chang, and Narayanan [32] introduced a technique to discover customer behavior changes in fuzzy association rules over time. Tsai and Shieh [33] first proposed a framework to observe the dynamic alternation of sequential patterns between two time periods to detect customer purchases and order changes.

Current research is limited to examinations of customer purchase behavior available in the literature. Little attention has been paid to mining the changes in individual travel behavior pattern changes from vehicle trajectory data.

## 3. Proposed Methodology

### 3.1. Problem Definition

Travel behavior spans multiple heterogeneous dimensions. In this paper, we focus on two critical dimensions—spatial and frequency—that characterize *where* and *how often* individuals travel, respectively. For a given individual, the two behavioral dimensions are typically correlated, but with some independence. Changes can occur to one dimension but not the other. For example, when a person moves to a new house, the spatial dimension of travel changes while its frequency stays the same.

**Figure 1:**
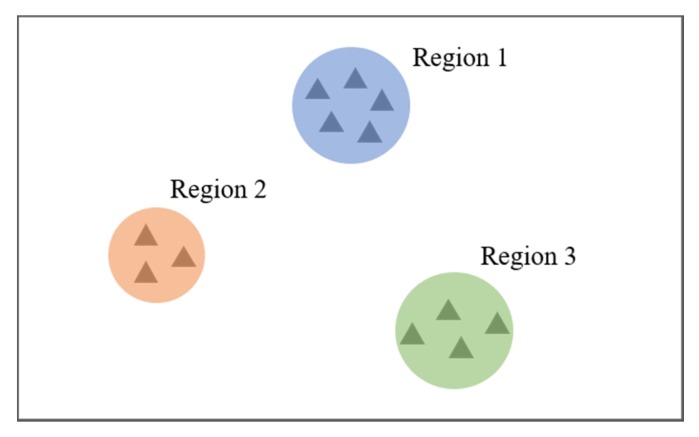
Illustration of spatial patterns.

**Definition** **1.** *Spatial patterns describe the distribution of destinations of trips an individual makes in space. In real life, a person usually parks their car in slightly different locations, even when going to the same intended destination. Furthermore, there exists a degree of drift in GPS/GNSS data collected from vehicles. Taking these factors into consideration, a region, rather than a precise location, is adopted to characterize the individual’s spatial pattern. An example is illustrated in Figure 1. In the figure, there are three regions represented by three different colors, indicating three regions frequently visited by the individual in their trips. A point in the region denotes a trip the individual makes.*

**Definition** **2.** *Frequency patterns describe the percentage of occurrence of a particular spatial pattern among all spatial patterns. For example, John made 100 trips last week: 50 to Region 1, 30 to Region 2, and 20 to Region 3; therefore, the frequencies of Regions 1, 2, and 3 are 50%, 30%, and 20%, respectively.*

### 3.2. Specification of Change Detection

Let us define the following notation to describe the process of extracting an individual’s travel pattern:

$D^{t1}$, $D^{t2}$: datasets at time periods *t*1, *t*2

$D^{total}$: *a* dataset formed by merging $D^{t1}$ and $D^{t2}$

$C^{t1}$, $C^{t2}$: discovered spatial pattern sets at time periods *t*1, *t*2

$C^{total}$: discovered spatial pattern set of $D^{total}$

$c_{i}^{t1}$, $c_{j}^{t2}$: each spatial pattern from the corresponding pattern set $C^{t1}$, $C^{t2}$,

where i=1, 2, …, j=1, 2, …

$c_{i}$: spatial pattern in $C^{total}$, where *i* = 1,2, …

$freq\left(c_{i}^{t1}\right)$, $freq\left(c_{j}^{t2}\right)$: frequency of $c_{i}^{t1}$, $c_{j}^{t2}$, where i=1, 2, …, j=1, 2, …

$F^{t1}$, $F^{t2}$: frequency set at time period *t*1, *t*2

**Figure 2:**
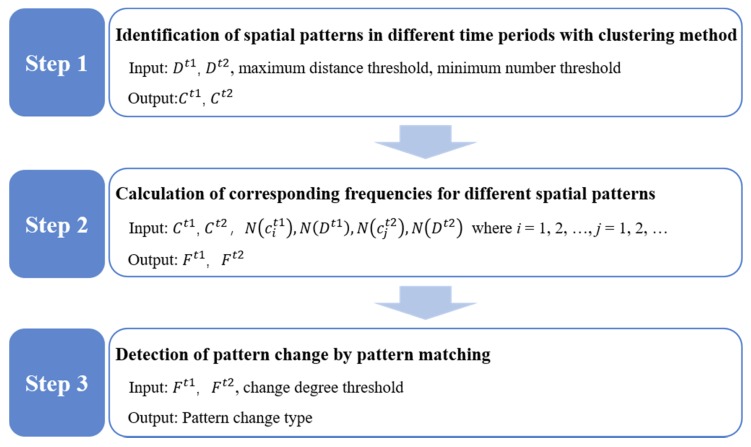
The framework of the change detection methodology.

The framework of the proposed methodology for the change detection problem, consisting of three steps, is shown in Figure 2.

Step 1 Identification of spatial patterns in different time periods using clustering.

Cars equipped with GPS/GNSS transmit the vehicle’s location to data centers at regular intervals. Data transmission starts when the vehicle is started and ends when the vehicle is turned off. The transmitted data usually contain the car’s latitude and longitude at different times. In this paper, we use the vehicle’s position at the end of every trip. Clustering techniques are important when it comes to extracting knowledge from a large amount of spatial data. Several clustering methods have become popular for extracting useful patterns from large-scale spatial data. DBSCAN is a pioneering density-based algorithm that can discover clusters of any arbitrary shape and size, even in databases containing noise and outliers. For the DBSCAN algorithm, we need a dataset, the maximum spatial distance value (*Eps*), and the minimum number of points within the *Eps* distance (*MinPts*) as inputs. The algorithm’s output is a set of clusters. If a point in the dataset does not belong to any cluster, it is marked as noise. In this paper, the DBSCAN method is adopted, and the identified clusters represent frequently visited regions.

**Figure 3:**
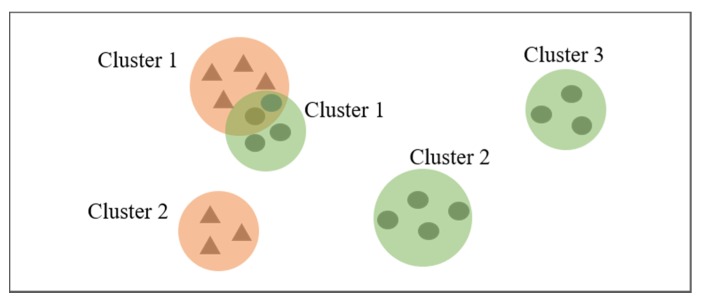
The clustering results of two datasets.

The most common approach to discovering changes between two datasets is to generate patterns from each dataset and directly compare the patterns using pattern matching. However, if DBSCAN is run on two datasets separately, a problem may arise, as illustrated in Figure 3. In the figure, the triangular points belong to $D^{t1}$, and the elliptic points belong to $D^{t2}$. The red regions represent clusters generated from $D^{t1}$, and the green regions represent the clusters generated from $D^{t2}$. As we can see, although Cluster 2 from $D^{t1}$ and $D^{t2}$ are marked with the same cluster label, they are two completely different clusters. Therefore, they cannot be compared directly; the relationship between clusters generated from different datasets must be evaluated, and the cluster labels should be updated. However, this process increases the complexity of solving the problem.

**Figure 4:**
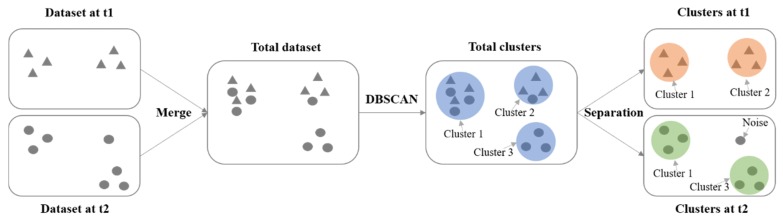
Clustering flowchart for two datasets.

In our research, we developed the simpler approach shown in Figure 4. First, the datasets generated at different time periods are merged into a total dataset. Then, DBSCAN is performed on the total dataset to detect clusters. Finally, for every cluster of the total dataset, the points belonging to different time periods are separated. If the number of the separated points is no smaller than *MinPts*, then the separated points are identified as a cluster of the original dataset, marked with the same cluster label as from the clusters from the total datasets; otherwise, they are marked as noise.

The pseudocode of the total calculation is shown in Algorithm 1:
**Algorithm 1** Pattern identification algorithm.**Procedure:** Pattern identification ($D^{t1}$*,*$D^{t2}$, *Eps*, *MinPts*)  1: combine $D^{t1}$ and $D^{t2}$ into a new dataset $D^{total}$
  2: DBSCAN ($D^{total}$, *Eps*, *MinPts*)  3: **for** every cluster *i*
**in**
$C^{total}$
**do**  4: **for** every point *j*
**in** cluster *i*
**do**  5: **if** point j ∈$D^{t1}$
**then**  6: mark the point *j*
**in**
$D^{t1}$ with label *i*  7: **else**  8: mark the point *j*
**in**
$D^{t2}$ with label *i*  9: **for** every cluster *m*
**in**
$C^{t1}$
**do**  10: **if** the number of points in cluster *m* < *MinPts*
**then**  11: mark the points in cluster *m* as noise  12: **for** every cluster *n*
**in**
$C^{t2}$
**do**  13: **if** the number of points in cluster *n* < *MinPts*
**then**  14: mark the points in cluster *n* as noise  
**return**
$C^{t1}$, $C^{t2}$


Step 2 Calculation of corresponding frequencies for different spatial patterns.

For our explanation of frequency, we briefly define the following notation:

$N\left(c_{i}^{t1}\right)$, $N\left(c_{j}^{t2}\right)$: Number of points in $c_{i}^{t1}$, $c_{j}^{t2}$, where i=1, 2, …, j=1, 2, …

$N\left(D^{t1}\right)$, $N\left(D^{t2}\right)$: Number of points in $D^{t1}$, $D^{t2}$

Now, we provide the calculation of $freq\left(c_{i}^{t1}\right)$, as shown in Equations (1) and (2):

(1) $$freq\left(r_{i}^{t1}\right)=\frac{N\left(c_{i}^{t1}\right)}{N\left(D^{t1}\right)}$$

(2) $$freq\left(r_{j}^{t2}\right)=\frac{N\left(c_{j}^{t2}\right)}{N\left(D^{t2}\right)}$$

The pseudocode of the total calculation is shown in Algorithm 2:
**Algorithm 2:** Frequency calculation algorithm.**Procedure** Frequency ($C^{t1},\;C^{t2},\;N\left(c_{i}^{t1}\right),\;N\left(D^{t1}\right),\;N\left(c_{j}^{t2}\right),\;N\left(D^{t2}\right)$)  1: **for** every cluster *i*
**in**
$C^{t1}$
**do**  2: calculate the frequency with $freq\left(c_{i}^{t1}\right)=\frac{N\left(c_{i}^{t1}\right)}{N\left(D^{t1}\right)}$
  3: **for** every cluster *j*
**in**
$C^{t2}$
**do**  4: calculate the frequency with $freq\left(c_{j}^{t1}\right)=\frac{N\left(c_{j}^{t2}\right)}{N\left(D^{t2}\right)}$
  **return**
$F^{t1}$, $F^{t2}$


Step 3 Detection of pattern changes by pattern matching.

**Figure 5:**
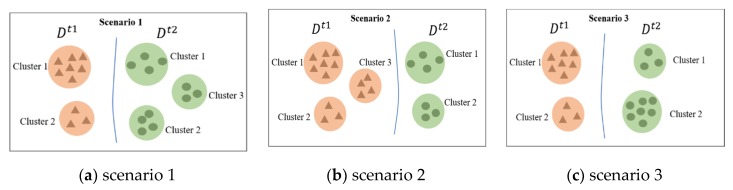
Three basic pattern-change scenarios.

When the clusters and corresponding frequencies of different datasets are compared, there are three basic pattern-change scenarios, as shown in Figure 5.

**Scenario** **1:** *In* Figure 5 *a, Cluster 3 arises in* $D^{t2}$ *but not in* $D^{t1}$ *. Scenario 2: Cluster 3 exists in* $D^{t1}$ *but not in* $D^{t2}$ *, as shown in* Figure 5 *b. Scenario 3: Clusters 1 and 2 exist in both* $D^{t1}$ *and* $D^{t2}$ *, but the frequencies of each cluster differ significantly between time periods t1 and t2, as shown in Figure 5c.*

In this study, we mark the newly arising clusters as *New* and disappearing clusters as *Vanished*. To better describe the change presented in Scenario 3, we propose a measure of the degree of change. The calculation of this measure is shown in Equation (3).

(3) $$R\left(c_{i}\right)=\left|\frac{freq\left(c_{i}^{t1}\right)-freq\left(c_{j}^{t2}\right)}{freq\left(c_{i}^{t1}\right)}\right|*100\%,\;i=j=1,2\ldots$$

where $R\left(c_{i}\right)$ represents the degree of change of $c_{i}$. A *threshold* for $R\left(c_{i}\right)$ should be defined manually. In other words, only if $R\left(c_{i}\right)$ is no less than the threshold should cluster $c_{i}$ be identified as a changed pattern; otherwise, it is identified as an unchanged pattern. If $c_{i}$ is a changed pattern, it is marked as *Increased* if $freq\left(c_{i}^{t1}\right)$ is less than $freq\left(c_{j}^{t2}\right)$; otherwise, it is marked as *Decreased*. Unchanged patterns are marked as *Unchanged*.

The pseudocode of the total calculation is shown in Algorithm 3:
**Algorithm 3:** Pattern-matching algorithm.**Procedure** Pattern match ($freq\left(c_{i}^{t1}\right)$, $freq\left(c_{j}^{t2}\right)$, change degree *threshold*)  1: **for**
$freq\left(c_{i}^{t1}\right)$
**in**
$F^{t1}$ and *f*$req\left(c_{j}^{t2}\right)$
**in**
$F^{t2}$ && *i* = *j*
**do**  2: **if**
$freq\left(c_{i}^{t1}\right)=0$
**then**  3: mark pattern $c_{i}$ as *New*
**break**  4: **if**
$freq\left(c_{j}^{t2}\right)=0$
**then**  5: mark pattern $c_{i}$ as *Vanished*
**break**  6: **if**
$freq\left(c_{i}^{t1}\right)=freq\left(c_{j}^{t2}\right)$
**then**  7: mark pattern $c_{i}$ as *Unchanged*
**break**  8: Calculate the change rate with $R\left(c_{i}\right)=\left|\frac{freq\left(c_{i}^{t1}\right)-freq\left(c_{j}^{t2}\right)}{freq\left(c_{i}^{t1}\right)}\right|*100\%$
  9: **if**
$R\left(c_{i}\right)\geq threshold$
**then**  10: **if**
*f*$req\left(c_{i}^{t1}\right)>freq\left(c_{j}^{t2}\right)$
**then**  11: mark pattern $c_{i}$ as *Decreased*
  12: **else**  13: mark pattern $c_{i}$ as *Increased*  14: **else**  15: mark pattern $c_{i}$ as *Unchanged*  **return**

## 4. Case Study

### 4.1. Data

To test the proposed change detection methodology, we used a dataset of user trajectory data collected by an automotive manufacturer that launched a smart connected electric car on 18 September 2018. The dataset contains user travel history, such as the date and time, speed, latitude, longitude, mileage, battery pack temperature, voltage, and current. To protect users’ privacy, we randomly selected three anonymized users. The attributes of parking time, latitude, and longitude were chosen, as shown in Table 1. To protect personal privacy, the last digit is replaced with “*”. There are 6760 rows in the dataset, ranging from 1 November 2018 to 31 October 2019. The rows that contain missing values were deleted in the data preprocessing stage. We prepared two datasets for every user to detect significant changes in their travel behavior. The time period of the first dataset was 1 November 2018–30 April 2019, which was marked as T1. The time period of the second dataset was 1 May 2019–31 October 2019, which was marked as T2.

### 4.2. Results and Discussion

**Figure 6:**
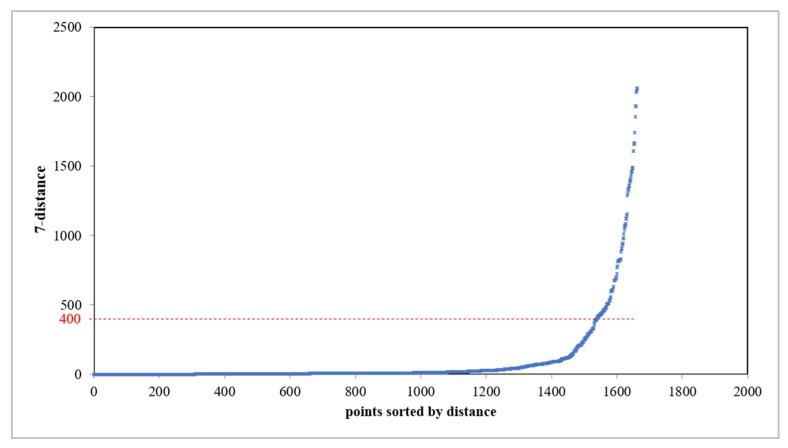
The illustration of the 7-distance plot.

We computed the datasets using the method mentioned above. For DBSCAN, referring to the existing research, MinPts≈ln(n), where n is the size of the database [34]. We analyzed the whole dataset; the range of n is about 1000–2500, so the value of MinPts was set to 7. Then, we observed a knee point in the 7-distance plot, as shown in Figure 6. The knee point is about 400. So, the parameter of Eps was set to 400 m. The threshold of the degree of change is 20%. 

**Figure 7:**
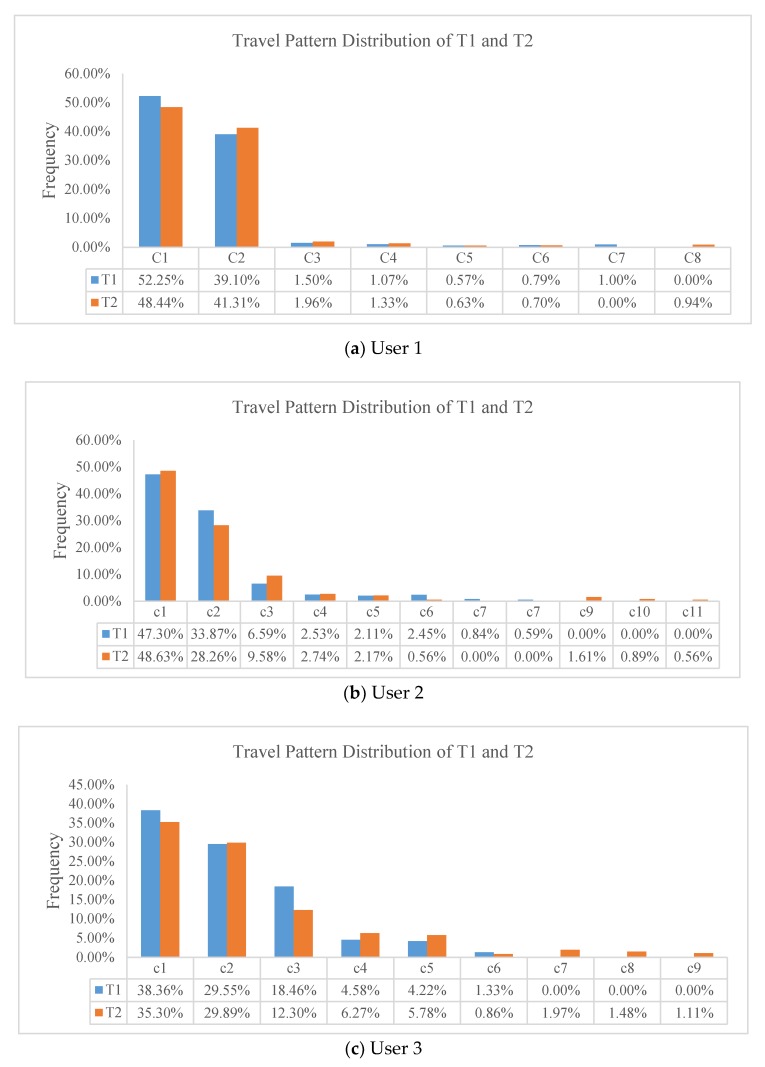
The distribution of travel patterns at T1 and T2 for the three users.

The results are shown in Table 2, Table 3 and Table 4 and in Figure 7.

**Figure 8:**
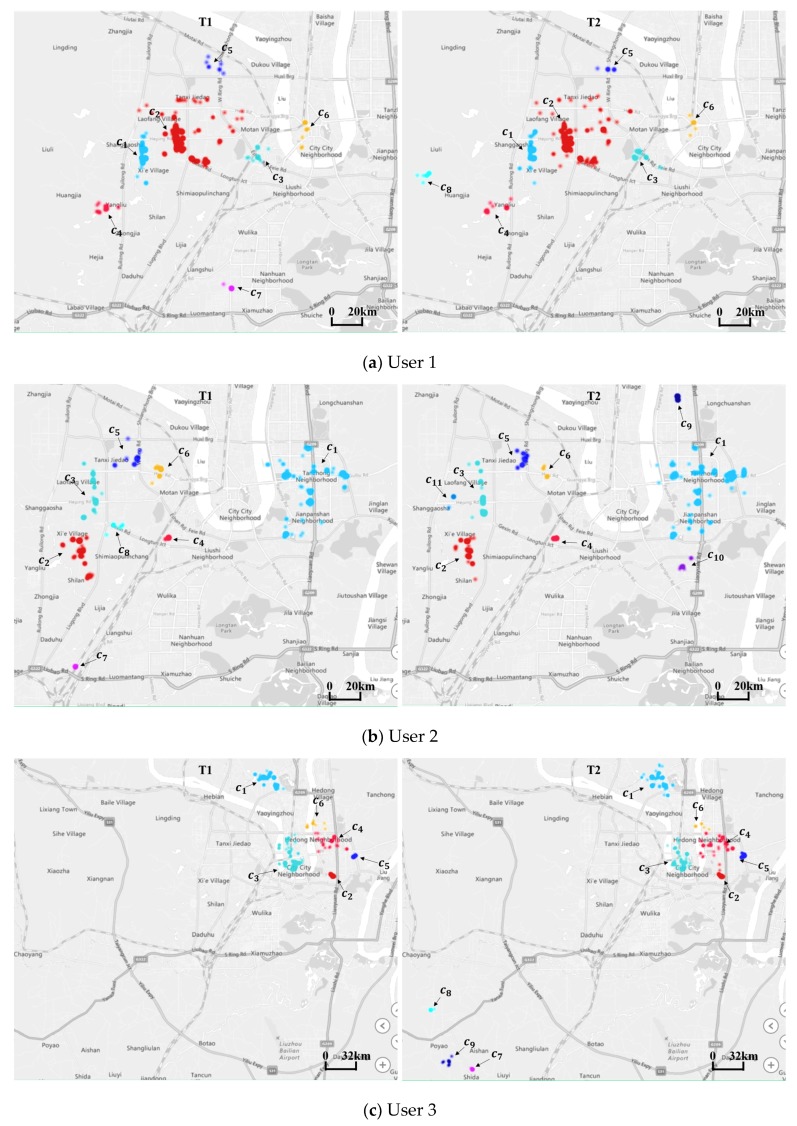
Mapped distributions of the three users’ travel patterns at T1 and T2.

As we can see in Figure 7, for every user, the top two patterns account for more than 70% of the total patterns and remain relatively stable. These two regions are very likely to represent the addresses of the user’s home and workplace. The sales department of the automotive manufacturer should study the distribution characteristics of such regions, which may contain their potential consumers, thus allowing them to advertise effectively there. From Table 2, Table 3 and Table 4, we find that new or vanished patterns occur for every user. In order to understand these patterns better, we display the regions on the map shown in Figure 8.

The patterns of $c_{7}$ and $c_{8}$ in Figure 8a; $c_{7}$, $c_{8}$, $c_{9}$, $c_{10}$, and $c_{11}$ in Figure 8b; and $c_{7}$, $c_{8}$, and $c_{9}$ in Figure 8c are new or vanished regions for Users 1, 2, and 3, respectively. These patterns are clearly distinct from others, especially for User 3, as shown in Figure 8c. The reasons behind these changes are probably various, for example: for someone used to eating in a restaurant, the place might have changed because of a change in tastes or the relocation of businesses; they may have friends or relatives who have been sick recently, and they need to go to the hospital to take care of them; as the weather changes, holiday travel may vary between parks, zoos, attractions, and indoor entertainment venues. Service providers need to consider whether they should build charging facilities in the new regions; automobile dealers may need to consider whether to invest in advertising in these places, as there may be potential consumer groups here. The causes and characteristics of this phenomenon deserve further study, combined with location semantic information. We also find increased and decreased patterns for each user: two ($c_{5}$, $c_{6}$) for User 1, two ($c_{3}$, $c_{6}$) for User 2, and four ($c_{3}$, $c_{4}$, $c_{5}$, $c_{6}$) for User 3, as shown in Table 2, Table 3 and Table 4. Although these regions account for no more than 20% of each user’s total patterns, they may represent the areas of the users’ daily shopping, dining, entertainment, and other activities. More valuable information could be yielded if the reasons for changes could be mined, and this information could generate commercial revenue. For example, LBS providers could adjust their recommendation strategies according to changes in travel preference.

## 5. Conclusions

In this paper, we present a methodology that detects changes in individual travel behavior from vehicle GPS data. Specifically, we consider two heterogeneous dimensions of individual travel behavior: spatial and frequency dimensions. We adopt a clustering method to identify travel patterns, and we then discover changes by comparing the patterns generated from datasets of different time periods. The methodology was tested using a dataset of electric vehicle users from a Chinese automaker. The results show that the proposed methodology is able to effectively detect pattern changes in individual travel behavior. However, the suggested methodology does have some limitations. First, user travel patterns contain not only spatial patterns but also temporal patterns, which are not included in this paper. The transition sequence and transition time between regions are also topics worthy of study in the future. Second, the reasons behind the pattern changes have not been explored. In future research, we plan to extend our methodology by considering more of the available data, especially the semantic location information, which can help us better understand users’ travel behavior. The ability to detect changes in individual travel patterns is important for urban planning, mobility management, vehicle function modification, and travel service improvement. The proposed methodology serves as a tool for understanding and quantifying the long-term dynamics of travel behavior. We believe that the change detection problem will become increasingly important as more data mining methods and applications are implemented.

## Figures and Tables

**Table 1:** Trajectory dataset of the three users.

User	Date/Time	Latitude	Longitude
1	1 November 2018 8:24	24.3131 *	109.3619 *
1	31 October 2019 18:40	24.3090 *	109.3505 *
2	1 November 2018 10:35	24.3027 *	109.3561 *
2	31 October 2019 21:45	24.3247 *	109.4509 *
3	1 November 2018 8:21	24.3712 *	109.3887 *
3	31 October 2019 19:44	24.3081 *	109.4373 *

**Table 2:** Change detection results for User 1.

Change Type	Patterns
New	$c_{8}$
Vanished	$c_{7}$
Increased	$c_{5}$
Decreased	$c_{6}$
Unchanged	$c_{1}$, $c_{2}$, $c_{3}$, $c_{4}$

**Table 3:** Change detection results for User 2.

Change Type	Patterns
New	$c_{9}$, $c_{10}$, $c_{11}$
Vanished	$c_{7}$, $c_{8}$
Increased	$c_{3}$
Decreased	$c_{6}$
Unchanged	$c_{1}$, $c_{2}$, $c_{4}$, $c_{5}$

**Table 4:** Change detection results for User 3.

Change Type	Patterns
New	$c_{7}$, $c_{8}$, $c_{9}$
Vanished	None
Increased	$c_{4}$, $c_{5}$
Decreased	$c_{3}$, $c_{6}$
Unchanged	$c_{1}$, $c_{2}$
